# Talk on the Materials and Methods Used in Taking Impressions

**Published:** 1887-05

**Authors:** W. H. Dorrance


### Talk on the Materials and Methods used in taking Impressions.

BY W. H. DORRANCE.

(Read before the Michigan State Dental Society, March 22, 1887.)

In the announcement a paper is promised on impressions, but I propose to occupy the time in a talk on impressions rather than a paper.

Inasmuch as the impression is the foundation in any piece ot prosthetic dentistry, it becomes a matter of importance that the operator should be acquainted with the material he has to use—with its capabilities and its faults. When we come to consider that we have in the mouth an object that is yielding in its texture, and is not uniformly hard or soft, it is a matter of moment that we are able to choose readily the material with which to take the impression.

Many teeth are saved that formerly would have been extracted.

The arch is expanded, and teeth are driven into place where formerly they would have been taken out. In order to do this work we must have good impressions to start with. We are in the habit of using plaster of Paris, yet I will venture to say that one out of ten impressioms will be fair, and the balance good for nothing; I may even say that one in twenty will be good and the balance good for nothing. It is nearly impossible for one to secure a perfect impression of the mouth for the reasons I have mentioned. We may take as a base for one proposition the inequality of the structure.

The material must be properly prepared. We must make use of a tray that shall come so nearly to the shape of the mouth that a minimum quantity of material shall be used. If it is plaster and too much is used you not only annoy the patient, but by pulling out of shape the overlying soft structure, the integrity of the impression is destroyed; so that we can easily see that a great deal of care must be used in the selection of a tray. Plaster of Paris will take as good an impression of the mouth as can be taken, if properly used. It is as we have it, Hydrated Calcium Sulphate. When mixed with water it immediately crystalizes

(though in another than its original form)—it sets, as we say. Each molecule of plaster takes two molecules of water in chemical union, and any excess of water is simply held mechanically. It is necessary to add the proper amount of water, as then the plaster will be stronger than it otherwise would, therefore, it is useless to go through the operation which is quite common, the beating of the plaster.

The proper method of mixing plaster is to take as small quantity of water as will be sufficient, dissolve in it the material with which to set it, and add the plaster by degrees until a little mound is built above the water; when this becomes saturated, pour off the excess of water and stir it only so much as to evenly mix it, as it is unwise to beat the plaster unless we wish to weaken it. What shall we use to set the plaster? Common salt is perhaps more used than anything else; but if you will use Sulphate of Potassium, I think you will be pleased with the result, as there is less change in the plaster in the use of this than with the other materials. What shall we separate our impressions with? I suppose shellac and oil are used more than anything else. Let those who have been using other means, try water alone until they get accustomed to it, and they will never use anything else. There are good reasons for not using oil or any greasy substance, one principal reason being sufficient. Water and oil do not agree, will not mix, and the oil is gathered into little pools rendering the model defective and its surface soiled. To separate the model from the impression by the use of water alone, thoroughly saturate the impression with cold water while the plaster is being mixed for the model. Blow off the water that stands on the surface and carefully pour in the plaster from one side so that the slight amount of water that stands on the surface may be carried out before it. When the model is thoroughly set, put the impression alone into boiling water for a moment, when it will readily leave the model. If you undertake to take a plaster impression when the teeth are all in the mouth you may find it difficult to remove the model. How will this be avoided? In the first place choose a tray as near the shape of the mouth as possible. The teeth should come down so as to touch the tray on either side, without resting in the center.

Take sufficient impression wax, prepared as for taking impressions, to fill the center of the tray, and with it carry the tray into the mouth so that the teeth rest upon its floor; remove and trim away either side from a broad base to an edge, so that the line through the center from front to back shall barely touch the palate. This will give two lines of weakness in the impression—the line of the teeth and the center of the arch, so that in removing the impression from the model the outer portion is brought out, and the two inner sections together breaking off at the lines of weakness and leaving the model intact. Should the impression leave the tray it is broken out of the mouth in the same way, the outward and downward motion for the outer portion and the inward motion for the palatine portion, and it will usually break into four pieces. In the selection of a tray for a lower impression, where the teeth are nearly all present, care should be taken that the tray does not rest upon the gums on the outside of the molars, but rest upon the teeth when in position, so as to give that line of weakness, hitherto referred to in connection with the upper impressions.

Now let us take up the consideration of other materials which have gradually been creeping into use in one way and another, known as modeling compounds. Some good, some bad, and some indifferent. The base of a majority of them is a fossil gum, usually gum kaurie, which is modified by the use of stearine and French chalk, to which is sometimes added a fragrant resin, and some coloring material. When properly prepared the ideal modeling compound, should soften at a comparatively low temperature, harden rapidly at the temperature of the mouth, at which temperature it should be somewhat elastic, and yet sufficiently rigid to retain its shape and integrity as an impression.

Here are samples of the leading modeling compounds acceptable to the profession in this country. This bears the stamp of Houghton & Reynolds. [Samples shown.] It softens at a low temperature, but it takes a long time to cool; then it has another serious fault, that while it is somewhat elastic, it has a serious tendency to shrink and crawl out of shape. These faults run through different grades of the same make, and render them worthless where accuracy is required. This sample of S. S. White's has a similar fault to a more limited extent; and I doubt not that many who have attempted to use these compounds, having met with these difficulties, have become thoroughly discouraged from the use of modeling compounds.

This sample [specimen] is Composition Magnifique, and is the only compound I have hitherto been able to obtain that may be said to be free from these faults. I am fortunate to-night in being able to introduce to your notice another modeling compound, lately sent me by its maker, Mr. Bolton, of Cardiff, Endland, which bids fair to prove itself superior in every respect to anything I have hitherto used. I think it is not too much to say that a familiar use of this material on the part of the profession in this country will bring them under an everlasting sense of obligation to its originator, Mr. Bolton.

The fact that the modeling compound or its equivalent is not so disagreeable to the patient and more agreeable to the operator than plaster should determine its more frequent and indeed habitual use for impressions in all but exceptional cases. To succeed with it one needs to become as familiar with its use and characteristics as they have already become with those of plaster. Now some hints in regard to the use of the modeling compound. Improperly used it has one serious fault, if the tray does not thoroughly support it and an excess is used, that excess pulls the impression out of shape as it falls over the posterior edge of the tray. So it follows that the tray must be shaped to the case, so as to support all the impression, and as little material used as may be necessary to take the impression properly.

If we would make a practice of forming a tray to suit each case we would secure better results with any material, but especially with this material we must have a tray that will just support it. This particular specimen (Mr. Bolton's) softens at about 120 Fareinheit in water, and the first thing to be observed in using it is that we need to have quite a volumn of water, say a quart, so that when once brought to the proper temperature it will retain the heat sufficiently long to enable us to complete the operation, and it should be borne in mind that an excess of heat will seriously injure any of the compounds, so it is best to remove the dish of water from the source of heat before putting in the compound. When thoroughly softened by the heat it should be molded into a smooth mass and shaped to the tray and placed in the mouth. But there are some principles involved that demand illustration.

[Illustrations on the board for the subsequent propositions.]

If we wish to take an impression of an object in low relief, as for instance a silver dollar, the impression on the material is applied in a direction of a line perpendicular to its surface. This principle must be followed in taking an impression of an arch in the mouth. We have in the teeth a surface of one direction, and in the arch a curve taking a different direction, and the matter becomes complicated, so we must choose a line of direction of pressure that will secure the best results on the surface we most need to use, which generally is the palatine line surface; therefore let the pressure be applied with a compound motion, so you will first carry the tray into the mouth, raise the posterior edges with a firm pressure against the posterior teeth so that the direction of pressure is perpendicular to the center of the curve. This forces the material to the center of the arch if when with a continuous motion the anterior portion of the tray is carried to place, when if we are using any of the plastic material for the impression that portion which is forced out by the edges of the teeth must be moulded to the outer portion of the ridge with a carefully complicated pressure, the lips covering the impression material. But now hold steadily in position, by the middle finger of the left hand in the center of the tray, until the compound is hard enough to bear removing. [While making this illustration and taking the impression before the Association the following hints were given.] The material should not be overheated as it renders it sticky and destroys its good qualities. You must bear in mind that in cold weather the composition cools in the air rapidly and its surface may need heating carefully over the blaze before introducing it into the mouth. This particular compound [Bolton's] softens at a lower temperature than the other varieties. It comes to the mouth relatively cool. If you are taking an impression for a regulating plate do not attempt to get too much of an impression, as for instance, a protruding arch. Seek to get an impression which you wish to use. Here in this patient's mouth we have a high arch. Supposing we apply the pressure directly upward, what will be the result? The mass of material will slide along the surface and be over the posterior edge of the tray, and when removed, instead of a sharp impression of the various features of the arch, it will look as though little fingers had been drawn along it. But we apply the pressure thus in a line perpendicular to the center ot the curve, and with a continuous motion gradually bring it off in front and we will find upon removing it that we have a good impression. I have said that a large amount of material tends to destroy the impression by the pulling of the soft structure out of position, or sometimes we wish to press portions of the arch out of their normal position, as for instance in an endentulous mouth, where excessive absorption of the bone leaves a flab of the gums. Now with the proper use of the plastic materials we can press this flab into a position that it will retain, when pressure is exerted in mastication. After an impression is removed from the mouth it should be washed in cold water, and the water driven from the surface by blowing upon filling with plaster for the model. All that is necessary in removing an impression from the model will be to soften it in warm water. If you will use the Modeling Compound long enough to become acquainted with it you will seldom find occasion to use plaster. The compound is much pleasanter to both patient and operator. You have a material that you can use over and over again without the least injury, and is as cleanly in its repeated use as napkins which are cleansed by washing.

I wish to speak of one more material, then relieve your patience. It is that material known as mouldine, Mellotte's mouldine, which is potter's clay moistened with glycerine instead of water. It is invaluable in crown and bridge work, as it takes a very sharp impression, in which the die can be directly cast. The material is furnished ready for use, with the proper trays and the fusible matter for dies, and should be in the hands of every prosthetic dentist.

